# Supplementary material for: Molecular characterization of nearshore baitfish populations in Bermuda to inform management
Source: PeerJ. 2019 Jul 4;7:e7244. doi: 10.7717/peerj.7244 (PMC6612424; doi:10.7717/peerj.7244)
Supplement: Appendix 1 — Detailed information regarding each specimen sequenced including sample ID, date of collection, Country, GPS coordinates of collection sites, forward and reverse primer names, and the assigned accession number for retrieving sequence data on the public genetic sequence database, Genbank (www.ncbi.nlm.nih.gov/Genbank). [file peerj-07-7244-s001.docx]

**Appendix 1. Sequence details.** Detailed information regarding each specimen sequenced including sample ID, date of collection, Country, GPS coordinates of collection sites, forward and reverse primer names, and the assigned accession number for retrieving sequence data on the public genetic sequence database, Genbank ([www.ncbi.nlm.nih.gov/Genbank)](http://www.ncbi.nlm.nih.gov/Genbank)).

| **Sequence ID** | **Collection date** | **Country** | **GPS Coordinates** | **Forward primer** | **Reverse primer** | **Accession**  **number** |
| --- | --- | --- | --- | --- | --- | --- |
| ACHO_BAMZ_210 | July/Aug_2017 | Bermuda | 32.323N_64.738W | C_FishF1t1 | C_FishR1t1 | MK871566 |
| ACHO_BAMZ_211 | July/Aug_2017 | Bermuda | 32.323N_64.738W | C_FishF1t1 | C_FishR1t1 | MK871576 |
| ACHO_BAMZ_212 | July/Aug_2017 | Bermuda | 32.323N_64.738W | C_FishF1t1 | C_FishR1t1 | MK871567 |
| ACHO_BAMZ_213 | July/Aug_2017 | Bermuda | 32.323N_64.738W | C_FishF1t1 | C_FishR1t1 | MK871578 |
| ACHO_BAMZ_214 | July/Aug_2017 | Bermuda | 32.323N_64.738W | C_FishF1t1 | C_FishR1t1 | MK871568 |
| ACHO_BAMZ_215 | July/Aug_2017 | Bermuda | 32.323N_64.738W | C_FishF1t1 | C_FishR1t1 | MK871569 |
| ACHO_BAMZ_216 | July/Aug_2017 | Bermuda | 32.323N_64.738W | C_FishF1t1 | C_FishR1t1 | MK871570 |
| ACHO_BAMZ_217 | July/Aug_2017 | Bermuda | 32.323N_64.738W | C_FishF1t1 | C_FishR1t1 | MK871571 |
| ACHO_EAST_1 | July/Aug_2017 | Bermuda | 32.249N_64.826W | C_FishF1t1 | C_FishR1t1 | MK871562 |
| ACHO_EAST_11 | July/Aug_2017 | Bermuda | 32.249N_64.826W | C_FishF1t1 | C_FishR1t1 | MK871561 |
| ACHO_EAST_15 | July/Aug_2017 | Bermuda | 32.249N_64.826W | C_FishF1t1 | C_FishR1t1 | MK871572 |
| ACHO_EAST_3 | July/Aug_2017 | Bermuda | 32.249N_64.826W | C_FishF1t1 | C_FishR1t1 | MK871563 |
| ACHO_EAST_4 | July/Aug_2017 | Bermuda | 32.249N_64.826W | C_FishF1t1 | C_FishR1t1 | MK871577 |
| ACHO_EAST_6 | July/Aug_2017 | Bermuda | 32.249N_64.826W | C_FishF1t1 | C_FishR1t1 | MK871580 |
| ACHO_EAST_7 | July/Aug_2017 | Bermuda | 32.249N_64.826W | C_FishF1t1 | C_FishR1t1 | MK871564 |
| ACHO_EAST_8 | July/Aug_2017 | Bermuda | 32.249N_64.826W | C_FishF1t1 | C_FishR1t1 | MK871579 |
| ACHO_WEST_148 | July/Aug_2017 | Bermuda | 32.256N_64.874W | C_FishF1t1 | C_FishR1t1 | MK871573 |
| ACHO_WEST_149 | July/Aug_2017 | Bermuda | 32.256N_64.874W | C_FishF1t1 | C_FishR1t1 | MK871574 |
| ACHO_WEST_150 | July/Aug_2017 | Bermuda | 32.256N_64.874W | C_FishF1t1 | C_FishR1t1 | MK871575 |
| ACHO_WEST_151 | July/Aug_2017 | Bermuda | 32.256N_64.874W | C_FishF1t1 | C_FishR1t1 | MK871565 |
| OPIO_BAMZ_21 | July/Aug_2017 | Bermuda | 32.323N_64.738W | C_FishF1t1 | C_FishR1t1 | MK871654 |
| OPIO_BAMZ_22 | July/Aug_2017 | Bermuda | 32.323N_64.738W | C_FishF1t1 | C_FishR1t1 | MK871648 |
| OPIO_BAMZ_23 | July/Aug_2017 | Bermuda | 32.323N_64.738W | C_FishF1t1 | C_FishR1t1 | MK871649 |
| OPIO_BAMZ_24 | July/Aug_2017 | Bermuda | 32.323N_64.738W | C_FishF1t1 | C_FishR1t1 | MK871650 |
| OPIO_BAMZ_25 | July/Aug_2017 | Bermuda | 32.323N_64.738W | C_FishF1t1 | C_FishR1t1 | MK871651 |
| OPIO_DEEP_115 | July/Aug_2017 | Bermuda | 32.306N_64.803W | C_FishF1t1 | C_FishR1t1 | MK871655 |
| OPIO_DEEP_116 | July/Aug_2017 | Bermuda | 32.306N_64.803W | C_FishF1t1 | C_FishR1t1 | MK871652 |
| OPIO_DEEP_117 | July/Aug_2017 | Bermuda | 32.306N_64.803W | C_FishF1t1 | C_FishR1t1 | MK871653 |
| HYPO_BAILEYS_134 | July/Aug_2017 | Bermuda | 32.349N_64.723W | C_FishF1t1 | C_FishR1t1 | MK871618 |
| HYPO_BAILEYS_135 | July/Aug_2017 | Bermuda | 32.349N_64.723W | C_FishF1t1 | C_FishR1t1 | MK871619 |
| HYPO_BAMZ_53 | July/Aug_2017 | Bermuda | 32.323N_64.738W | C_FishF1t1 | C_FishR1t1 | MK871611 |
| HYPO_BAMZ_55 | July/Aug_2017 | Bermuda | 32.323N_64.738W | C_FishF1t1 | C_FishR1t1 | MK871612 |
| HYPO_BAMZ_57 | July/Aug_2017 | Bermuda | 32.323N_64.738W | C_FishF1t1 | C_FishR1t1 | MK871620 |
| HYPO_BAMZ_59 | July/Aug_2017 | Bermuda | 32.323N_64.738W | C_FishF1t1 | C_FishR1t1 | MK871621 |
| HYPO_BAMZ_60 | July/Aug_2017 | Bermuda | 32.323N_64.738W | C_FishF1t1 | C_FishR1t1 | MK871622 |
| HYPO_BAMZ_61 | July/Aug_2017 | Bermuda | 32.323N_64.738W | C_FishF1t1 | C_FishR1t1 | MK871608 |
| HYPO_BAMZ_95 | July/Aug_2017 | Bermuda | 32.323N_64.738W | C_FishF1t1 | C_FishR1t1 | MK871613 |
| HYPO_BAMZ_96 | July/Aug_2017 | Bermuda | 32.323N_64.738W | C_FishF1t1 | C_FishR1t1 | MK871614 |
| HYPO_FRANKS_50 | July/Aug_2017 | Bermuda | 32.255N_64.857W | C_FishF1t1 | C_FishR1t1 | MK871623 |
| HYPO_FRANKS_51 | July/Aug_2017 | Bermuda | 32.255N_64.857W | C_FishF1t1 | C_FishR1t1 | MK871624 |
| HYPO_FRANKS_52 | July/Aug_2017 | Bermuda | 32.255N_64.857W | C_FishF1t1 | C_FishR1t1 | MK871625 |
| HYPO_FRANKS_9 | July/Aug_2017 | Bermuda | 32.255N_64.857W | C_FishF1t1 | C_FishR1t1 | MK871626 |
| HYPO_FRANKS_9B | July/Aug_2017 | Bermuda | 32.255N_64.857W | C_FishF1t1 | C_FishR1t1 | MK871627 |
| HYPO_FRANKS_9C | July/Aug_2017 | Bermuda | 32.255N_64.857W | C_FishF1t1 | C_FishR1t1 | MK871615 |
| HYPO_FRANKS_9D | July/Aug_2017 | Bermuda | 32.255N_64.857W | C_FishF1t1 | C_FishR1t1 | MK871628 |
| HYPO_WEST_140 | July/Aug_2017 | Bermuda | 32.364N_64.714W | C_FishF1t1 | C_FishR1t1 | MK871616 |
| HYPO_WEST_141 | July/Aug_2017 | Bermuda | 32.364N_64.714W | C_FishF1t1 | C_FishR1t1 | MK871629 |
| HYPO_WEST_145 | July/Aug_2017 | Bermuda | 32.364N_64.714W | C_FishF1t1 | C_FishR1t1 | MK871630 |
| HYPO_WEST_155 | July/Aug_2017 | Bermuda | 32.364N_64.714W | C_FishF1t1 | C_FishR1t1 | MK871609 |
| HYPO_WEST_156 | July/Aug_2017 | Bermuda | 32.364N_64.714W | C_FishF1t1 | C_FishR1t1 | MK871631 |
| HYPO_WEST_158 | July/Aug_2017 | Bermuda | 32.364N_64.714W | C_FishF1t1 | C_FishR1t1 | MK871632 |
| HYPO_WEST_159 | July/Aug_2017 | Bermuda | 32.364N_64.714W | C_FishF1t1 | C_FishR1t1 | MK871610 |
| HYPO_WEST_160 | July/Aug_2017 | Bermuda | 32.364N_64.714W | C_FishF1t1 | C_FishR1t1 | MK871617 |
| SARD_BAMZ_26 | July/Aug_2017 | Bermuda | 32.323N_64.738W | C_FishF1t1 | C_FishR1t1 | MK871646 |
| SARD_BAMZ_27 | July/Aug_2017 | Bermuda | 32.323N_64.738W | C_FishF1t1 | C_FishR1t1 | MK871647 |
| HAHU_BAMZ_81 | July/Aug_2017 | Bermuda | 32.323N_64.738W | C_FishF1t1 | C_FishR1t1 | MK871641 |
| HAHU_BAMZ_82 | July/Aug_2017 | Bermuda | 32.323N_64.738W | C_FishF1t1 | C_FishR1t1 | MK871642 |
| HAHU_BAMZ_83 | July/Aug_2017 | Bermuda | 32.323N_64.738W | C_FishF1t1 | C_FishR1t1 | MK871633 |
| HAHU_BAMZ_85 | July/Aug_2017 | Bermuda | 32.323N_64.738W | C_FishF1t1 | C_FishR1t1 | MK871640 |
| HAHU_BAMZ_86 | July/Aug_2017 | Bermuda | 32.323N_64.738W | C_FishF1t1 | C_FishR1t1 | MK871634 |
| HAHU_BAMZ_87 | July/Aug_2017 | Bermuda | 32.323N_64.738W | C_FishF1t1 | C_FishR1t1 | MK871639 |
| HAHU_CO_1 | July/Aug_2017 | Bermuda | 32.359N_64.715W | C_FishF1t1 | C_FishR1t1 | MK871638 |
| HAHU_CO_3 | July/Aug_2017 | Bermuda | 32.359N_64.715W | C_FishF1t1 | C_FishR1t1 | MK871637 |
| HAHU_CO_5 | July/Aug_2017 | Bermuda | 32.359N_64.715W | C_FishF1t1 | C_FishR1t1 | MK871636 |
| HAHU_CO_6 | July/Aug_2017 | Bermuda | 32.359N_64.715W | C_FishF1t1 | C_FishR1t1 | MK871635 |
| JLAM_BAMZ_171 | July/Aug_2017 | Bermuda | 32.323N_64.738W | C_FishF1t1 | C_FishR1t1 | MK871588 |
| JLAM_BAMZ_172 | July/Aug_2017 | Bermuda | 32.323N_64.738W | C_FishF1t1 | C_FishR1t1 | MK871593 |
| JLAM_BAMZ_173 | July/Aug_2017 | Bermuda | 32.323N_64.738W | C_FishF1t1 | C_FishR1t1 | MK871594 |
| JLAM_BAMZ_174 | July/Aug_2017 | Bermuda | 32.323N_64.738W | C_FishF1t1 | C_FishR1t1 | MK871582 |
| JLAM_BAMZ_175 | July/Aug_2017 | Bermuda | 32.323N_64.738W | C_FishF1t1 | C_FishR1t1 | MK871592 |
| JLAM_BAMZ_176 | July/Aug_2017 | Bermuda | 32.323N_64.738W | C_FishF1t1 | C_FishR1t1 | MK871595 |
| JLAM_BAMZ_177 | July/Aug_2017 | Bermuda | 32.323N_64.738W | C_FishF1t1 | C_FishR1t1 | MK871589 |
| JLAM_BAMZ_178 | July/Aug_2017 | Bermuda | 32.323N_64.738W | C_FishF1t1 | C_FishR1t1 | MK871590 |
| JLAM_CO_161 | July/Aug_2017 | Bermuda | 32.359N_64.715W | C_FishF1t1 | C_FishR1t1 | MK871583 |
| JLAM_CO_162 | July/Aug_2017 | Bermuda | 32.359N_64.715W | C_FishF1t1 | C_FishR1t1 | MK871596 |
| JLAM_CO_163 | July/Aug_2017 | Bermuda | 32.359N_64.715W | C_FishF1t1 | C_FishR1t1 | MK871597 |
| JLAM_CO_164 | July/Aug_2017 | Bermuda | 32.359N_64.715W | C_FishF1t1 | C_FishR1t1 | MK871598 |
| JLAM_CO_165 | July/Aug_2017 | Bermuda | 32.359N_64.715W | C_FishF1t1 | C_FishR1t1 | MK871591 |
| JLAM_CO_166 | July/Aug_2017 | Bermuda | 32.359N_64.715W | C_FishF1t1 | C_FishR1t1 | MK871599 |
| JLAM_CO_167 | July/Aug_2017 | Bermuda | 32.359N_64.715W | C_FishF1t1 | C_FishR1t1 | MK871584 |
| JLAM_CO_168 | July/Aug_2017 | Bermuda | 32.359N_64.715W | C_FishF1t1 | C_FishR1t1 | MK871600 |
| JLAM_CO_4 | July/Aug_2017 | Bermuda | 32.359N_64.715W | C_FishF1t1 | C_FishR1t1 | MK871585 |
| JLAM_FRANKS_49 | July/Aug_2017 | Bermuda | 32.255N_64.857W | C_FishF1t1 | C_FishR1t1 | MK871601 |
| JLAM_SOUTH_201 | July/Aug_2017 | Bermuda | 32.275N_64.774W | C_FishF1t1 | C_FishR1t1 | MK871602 |
| JLAM_SOUTH_202 | July/Aug_2017 | Bermuda | 32.275N_64.774W | C_FishF1t1 | C_FishR1t1 | MK871586 |
| JLAM_SOUTH_203 | July/Aug_2017 | Bermuda | 32.275N_64.774W | C_FishF1t1 | C_FishR1t1 | MK871581 |
| JLAM_SOUTH_204 | July/Aug_2017 | Bermuda | 32.275N_64.774W | C_FishF1t1 | C_FishR1t1 | MK871587 |
| JLAM_SOUTH_205 | July/Aug_2017 | Bermuda | 32.275N_64.774W | C_FishF1t1 | C_FishR1t1 | MK871603 |
| JLAM_SOUTH_206 | July/Aug_2017 | Bermuda | 32.275N_64.774W | C_FishF1t1 | C_FishR1t1 | MK871604 |
| JLAM_SOUTH_207 | July/Aug_2017 | Bermuda | 32.275N_64.774W | C_FishF1t1 | C_FishR1t1 | MK871605 |
| JLAM_SOUTH_208 | July/Aug_2017 | Bermuda | 32.275N_64.774W | C_FishF1t1 | C_FishR1t1 | MK871606 |
| JLAM_TURTLE_255 | July/Aug_2017 | Bermuda | 32.354N_64.657W | C_FishF1t1 | C_FishR1t1 | MK871607 |
